# Surgical results of internal limiting membrane flap inversion and internal limiting membrane peeling for macular hole

**DOI:** 10.1371/journal.pone.0203789

**Published:** 2018-09-13

**Authors:** Hiroya Ota, Hiroshi Kunikata, Naoko Aizawa, Toru Nakazawa

**Affiliations:** 1 Department of Ophthalmology, Tohoku University Graduate School of Medicine, Sendai, Japan; 2 Department of Retinal Disease Control, Tohoku University Graduate School of Medicine, Sendai, Japan; 3 Department of Advanced Ophthalmic Medicine, Tohoku University Graduate School of Medicine, Sendai, Japan; 4 Department of Ophthalmic Imaging and Information Analytics, Tohoku University Graduate School of Medicine, Sendai, Japan; Massachusetts Eye & Ear Infirmary, Harvard Medical School, UNITED STATES

## Abstract

**Purpose:**

The internal limiting membrane (ILM) is a normal part of the retina, and the outcomes of ILM removal have not been fully investigated. ILM flap inversion is a recently developed technique that increases the success rate of macular hole (MH) surgery. Thus, we compared the anatomical closure rate and visual outcome in patients undergoing microincision vitrectomy surgery (MIVS) with ILM flap inversion or conventional ILM peeling for the treatment of MH.

**Methods:**

The medical records of 90 eyes with MH were reviewed retrospectively. The patients were classified into two groups based on MIVS procedure (group 1: ILM flap inversion, 46 eyes; group 2: ILM peeling, 44 eyes).

**Results:**

Preoperative characteristics were similar in the two groups, and there were no significant differences in 1 month- or 6 month-postoperative VA between the groups (*P* = 0.25 and *P* = 0.42, respectively). However, the surgical success rate was significantly higher in group 1 than group 2 (*P* = 0.04; 46/46: 100% and 41/44: 93%, respectively). Multiple regression analysis revealed that axial length and MH diameter were independent factors predicting 6-month postoperative BCVA in group 2 (*P* = 0.001 and *P* = 0.03, respectively), and that MH diameter was an independent factor predicting 6-month postoperative VA in group 1 (*P* = 0.03). Logistic regression analysis revealed that axial length (OR = 2.11; *P* = 0.02; area under the curve: 0.94; cut off score: 28.4 mm) was an independent factor indicating surgical failure in group 2.

**Conclusion:**

Our results suggest that MIVS with ILM flap inversion might be best suited to treat MH, particularly in patients with high myopia.

## Introduction

Macular hole (MH) can cause severe visual disturbance, but remarkable progress has been achieved in surgical treatment for eyes with this condition. Vitrectomy with internal limiting membrane (ILM) peeling allows a very high success rate for MH closure (approaching 95%),[1–4] even when less-invasive surgical interventions are used, such as microincision vitrectomy surgery (MIVS). However, there are a small number of eyes, especially those with a large or long-standing MH, in which conventional ILM peeling is unsuccessful. This often leads to additional operations and a poor visual outcome.[5–7]

The ILM flap inversion technique,[8] in which the MH is covered with a rolled segment of the peeled ILM, has been recently developed to overcome the challenge of treating eyes with difficult MHs. This technique can prevent the MH from having a postoperative flat-open appearance with bare retinal pigment epithelium, and improves both the functional and anatomic outcomes. Generally, this technique is recommended for eyes with large MHs, i.e., with an MH diameter greater than 400 μm,[8, 9] or for myopic eyes with MH.[10, 11] However, to the best of our knowledge, there are no current reports investigating the potential of clinical parameters, including MH diameter and axial length, to serve as prognostic indicators of MH closure and visual outcome. ILM flap inversion is relatively difficult for even experienced surgeons to perform, and thus it is important to establish clear criteria indicating the need for ILM flap inversion or conventional ILM peeling.

Thus, the current study sought to establish criteria indicating the need for ILM flap inversion. At our institution, MIVS with ILM flap inversion has been used to treat MH since 2012. Thus, we performed a retrospective investigation of the medical records of these patients, and compared them with the records of patients who underwent conventional ILM peeling after 2012. We evaluated various preoperative findings in these two groups, including MH diameter and axial length, and compared them with the anatomical closure rate and visual outcome.

## Patient and methods

### Subjects

This was a retrospective, interventional case series. We analyzed the medical records of 90 eyes that underwent MIVS at Tohoku University Hospital, including 46 consecutive eyes that underwent ILM flap inversion between June 2012 and June 2016 (group 1) and 44 consecutive eyes that underwent conventional ILM peeling in the same period (group 2). All surgeries were performed by one experienced surgeon (H.K.), who began to use the ILM flap inversion technique in June 2012. The surgeon chose the ILM flap procedure based on MH size (diameter > 400 micrometers), in addition to other factors such as MH duration (symptomatic duration > 3 months), or based on his discretion. Eyes were excluded if they had prior vitreoretinal surgery, proliferative retinopathy, retinal vascular disease, traumatic MH, retinal detachment due to MH, lacked clinical data for OCT and axial length, or if the patient was an adolescent. After the purpose and procedures of the operation were explained, informed consent was obtained from all patients. The procedures conformed to the tenets of the Declaration of Helsinki, and the study was approved by the institutional review board of Tohoku University Graduate School of Medicine.

### Surgical procedure

All surgeries were performed under local anesthesia and used either 25G or 27GMIVS. First, an infusion cannula was inserted through the inferotemporal sclera followed by the insertion of two cannulas through superotemporal and superonasal sites, which were kept closed until the vitrectomy began. Next, a corneal tunnel incision was made to perform phacoemulsification, aspiration, and intraocular lens implantation, if needed, before the vitrectomy. Patients with phakic eyes underwent MIVS combined with cataract surgery, and patients with pseudophakic eyes underwent only MIVS. After resecting the vitreal core, about 4 mg of triamcinolone acetonide (TA) was injected into the vitreous cavity to determine whether a posterior vitreous detachment (PVD) was present. If a PVD was not present, a PVD was created. After creating a PVD and removing peripheral residual gel, the ILM flap inversion was performed in group 1,[8] and the ILM was completely peeled and removed in group 2, assisted by TA in both groups.[12] At a minimum, efforts were made to remove the ILM from the area of the vascular arcade. Finally, fluid-air exchange was performed at the end of surgery.

### Measurement of clinical characteristics

All patients underwent a complete ocular examination 6 months after surgery. The analyzed data included age, sex, laterality, duration of symptoms, surgical procedure, preoperative intraocular pressure (IOP), axial length, MH diameter, MH stage, and rate of MH closure. Axial length was measured with an optical biometer (IOL Master; Carl Zeiss Meditec, Oberkochen, Germany). MH diameter was defined as the minimum distance between the open MH and a line parallel to the retinal pigment epithelium, and was measured with optical coherence tomography (OCT) (CIRRUS HD-OCT; Carl Zeiss Meditec, Oberkochen, Germany) throughout the follow-up period. MH stage was classified according to the Gass classification method.[13] MH closure was determined with both a slit-lamp examination and OCT. Preoperative, 1 month-postoperative and 6 month-postoperative decimal best-corrected VA was measured with the Landolt C visual acuity chart. Decimal acuity values were converted to logarithm of the minimal angle of resolution (log MAR) units. The macula was also examined with OCT in all patients preoperatively, and 1- and 6-months postoperatively; IS/OS disruption and ELM disruption were also evaluated at these time points.

### Statistical analyses

All statistical analyses were performed with JMP software (Pro version 11.0.0, SAS Institute Japan Inc., Tokyo, Japan). The data are presented as means ± standard error of the mean. An unpaired t-test was used to compare differences in background characteristics between the groups. The chi-square test was used for frequency data on sex, laterality, MH closure and MH duration ≥ 3 months. The Wilcoxon signed-ranks test was used for to analyze time-course differences in VA in the groups. A multiple linear regression analysis was performed in each group to determine independent variables affecting 6 month-postoperative VA. A logistic regression analysis was performed to determine independent variables contributing to surgical failure, i.e., an unclosed MH after MIVS. The receiver operating characteristic curve (ROC) for axial length was plotted to determine the optimum cut-off point, and the area under the ROC curve (AUC) was calculated to determine the power of discrimination between surgical success and failure. Spearman’s rank correlation test was used to estimate the relationships between axial length with 6 month-postoperative VA. The significance level was set at *P* < 0.05.

## Results

Ninety Japanese patients with MH (31 male, 59 female) were recruited for this study. The patients were classified into two groups based on surgical procedure (group 1: ILM flap inversion, 46 eyes; group 2: conventional ILM peeling, 44 eyes). The clinical characteristics of the MH patients in each group are shown in Table 1. The ratio between new cases of MH (MH duration < 3 months) and long-standing cases of MH (MH duration > 3 months) was 22:24 in group 1 and 31:13 in group 2, a statistically significant difference (P = 0.03). There were no other significant differences in any preoperative characteristics between the groups. Six of 46 eyes (13%) in group 1 and 8 of 44 eyes in group 2 (18%) had high myopia (axial length ≥ 26.0 mm or spherical equivalent ≤ -6.0D), which was not a significant difference (P = 0.70).

**Table 1:** Characteristics of each group.

	Group 1 (inverted ILM flap)	Group 2 (ILM peeling)	*P* value
Number of eyes	46	44	
Age (years)	65.6 ± 8.1	65.5 ± 11.3	0.97
Sex (F:M)	27 : 19	32 : 12	0.16
Laterality (R:L)	18 : 28	20 : 24	0.54
MH duration (months)	7.8 ± 18.3	7.9 ± 36.4	0.98
Procedure			0.23
Vitrectomy with cataract surgery	40	34	
Only vitrectomy	6	10	
IOP (mmHg)	14.7 ± 4.0	15.0 ± 3.5	0.53
Axial length (mm)	24.2 ± 1.8	24.4 ± 2.3	0.72
MH diameter (μm)	491.5 ± 135.9	465.9 ± 129.5	0.36
MH stage (1:2:3:4)	0:5:5:36	1:7:5:31	0.56
MH closure success: failure	46 : 0	41 : 3	0.04
VA (logMAR)			
Preoperative	0.8 ± 0.3	0.7 ± 0.3	0.06
1M-postoperative	0.5 ± 0.3	0.4 ± 0.4	0.25
6M-postoperative	0.4 ± 0.3	0.3 ± 0.3	0.42
OCT findings			
1M-postoperative IS/OS disruption (n, %)	41, 89	33, 75	0.13
6M-postoperative IS/OS disruption (n, %)	14, 30	22, 50	0.80
1M-postoperative ELM disruption (n, %)	26, 57	16, 36	0.08
6M-postoperative ELM disruption (n, %)	7, 15	12, 27	0.72

ILM = internal limiting membrane, MH = macular hole, IOP = intraocular pressure, VA = visual acuity, logMAR = logarithm of minimum angle of resolution, OCT = optical coherence tomography, IS/OS = photoreceptor inner and outer segment, ELM = external limiting membrane

Three of the 90 eyes, all in group 2, failed to achieve MH closure after MIVS. There were no significant differences in 1 month- or 6 month-postoperative VA between the groups (*P* = 0.25 and *P* = 0.42, respectively). There were also no significant differences in postoperative OCT findings at 1 and 6 months postoperatively, including in photoreceptor inner and outer segment (IS/OS) disruption (*P* = 0.13 and *P* = 0.80, respectively) and external limiting membrane (ELM) disruption (*P* = 0.08 and *P* = 0.72, respectively).

**Fig 1:**
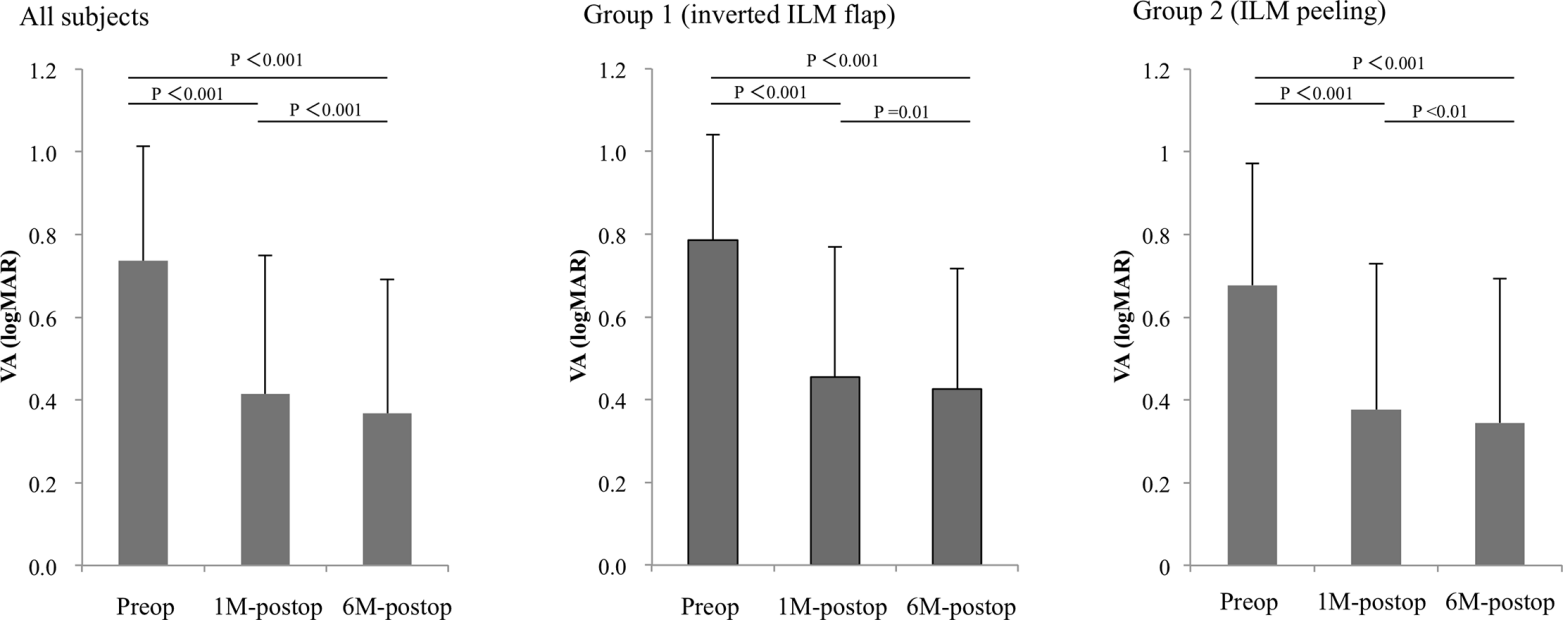
Time course of postoperative improvement in visual acuity (VA). Left, center, right: Preoperative, one month-postoperative, and six month-postoperative VA in all subjects, subjects who underwent internal limiting membrane (ILM) flap inversion, and subjects who underwent conventional ILM peeling, respectively. One-month postoperative VA was significantly better than preoperative VA in all subjects and in both groups individually. Furthermore, six-month postoperative VA was significantly better than preoperative VA and 1-month postoperative VA in all subjects and in both groups individually (better than *P* < 0.05 for all values).

Changes in visual acuity (VA) are shown in Fig 1. One-month postoperative VA was significantly better than preoperative VA in all subjects (*P* < 0.001). Six-month postoperative VA was also significantly better than preoperative VA and 1-month postoperative VA in all subjects (*P* < 0.001). These results were similar in both groups when they were examined individually (better than *P* < 0.05 for all values).

Multiple regression analysis revealed that MH diameter was an independent factor predicting 6-month postoperative VA in group 1 (*P* = 0.03; Table 2), and that axial length and MH diameter were independent factors predicting 6-month postoperative VA in group 2 (*P* = 0.001 and *P* = 0.03, respectively; Table 3).

**Table 2:** Multiple regression analysis of independent factors associated with 6-month postoperative VA in group 1 (inverted ILM flap).

Variables			
Dependent	Independent	β	*P* value
6M-postoperative VA	Continuous variables		
	Age (years)	0.37	0.08
	Axial length (mm)	0.38	0.08
	MH diameter (μm)	0.44	0.03
	Categorical variables		
	MH stage 4	-0.06	0.74

**Table 3:** Multiple regression analysis of independent factors associated with 6-month postoperative VA in group 2 (ILM peeling).

Variables			
Dependent	Independent	β	*P* value
6M-postoperative VA	Continuous variables		
	Age (years)	0.32	0.06
	Axial length (mm)	0.61	0.001
	MH diameter (μm)	0.39	0.03
	Categorical variables		
	MH stage 4	-0.06	0.73

Logistic regression analysis revealed that axial length (odds ratio (OR) = 2.11; 95% confidence interval (CI): 1.11–4.01; *P* = 0.02; Table 4), was an independent factor indicating surgical failure in group 2, while age, MH diameter, and an MH stage of 4 were not (Table 4).

**Table 4:** Logistic regression analysis of factors predicting surgical failure in group 2 (ILM peeling).

Dependent	Independent	Odds ratio	95% CI	*P* value
Surgical failure	Continuous variables			
	Age (years)	0.90	0.71–1.15	0.40
	Axial length (mm)	2.11	1.11–4.01	0.02
	MH diameter (μm)	1.00	0.98–1.02	0.77
	Categorical variables			
	MH stage 4	0.06	0.00–5.55	0.23

**Fig 2:**
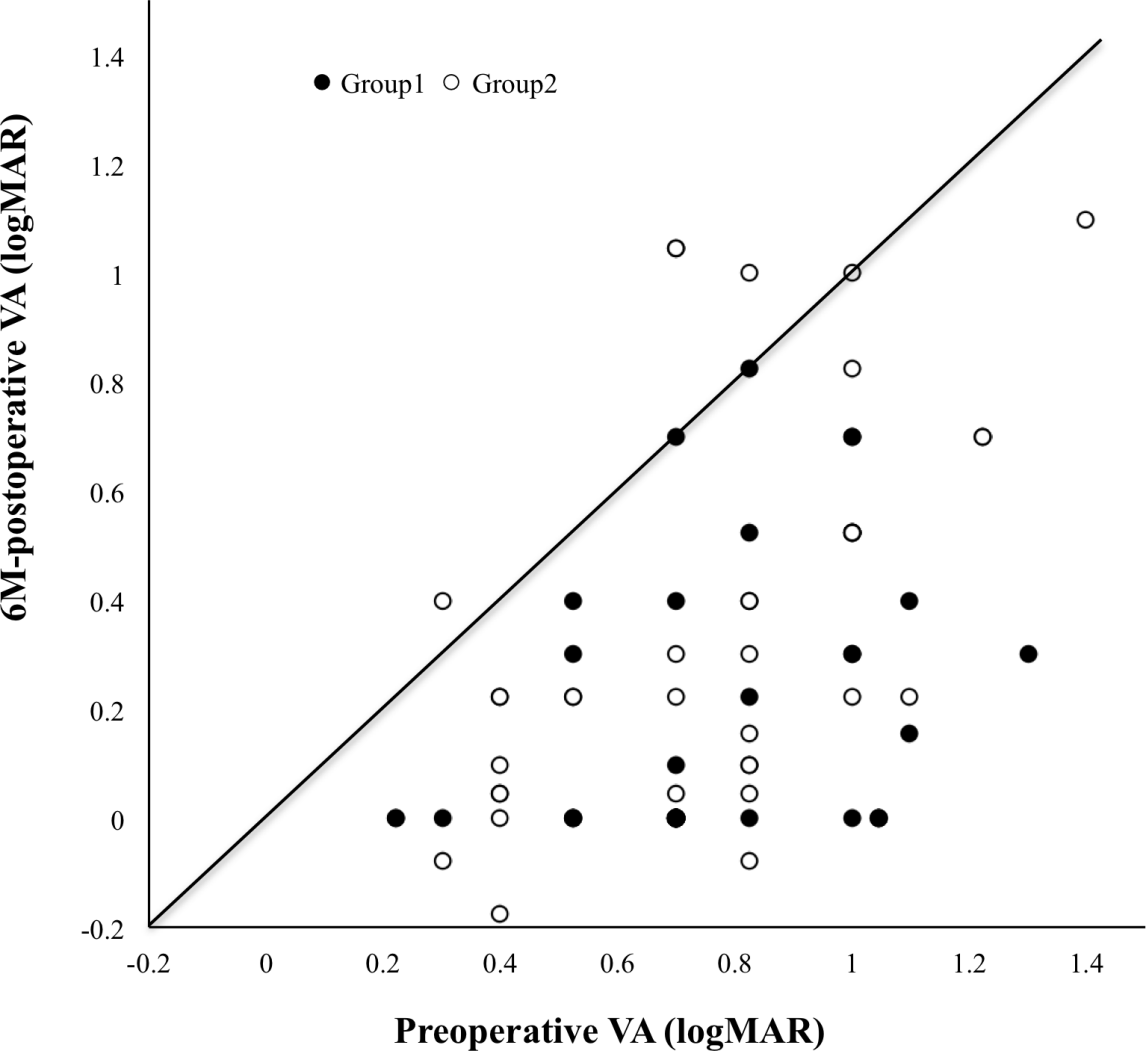
Scatter plot diagram of pre- and post-op VA in both groups. The open circles and closed circles show group 1 (ILM flap inversion, 46 eyes) and group 2 (ILM peeling, 44 eyes), respectively. There were no eyes with decreased visual acuity after surgery in group 1. However, there were 3 eyes with decreased visual acuity after surgery in group 2.

Fig 2 shows a scatter plot diagram of preoperative and postoperative VA for both groups. There were no eyes with decreased visual acuity after surgery in group 1. However, there were 3 eyes with decreased visual acuity after surgery in group 2.

**Fig 3:**
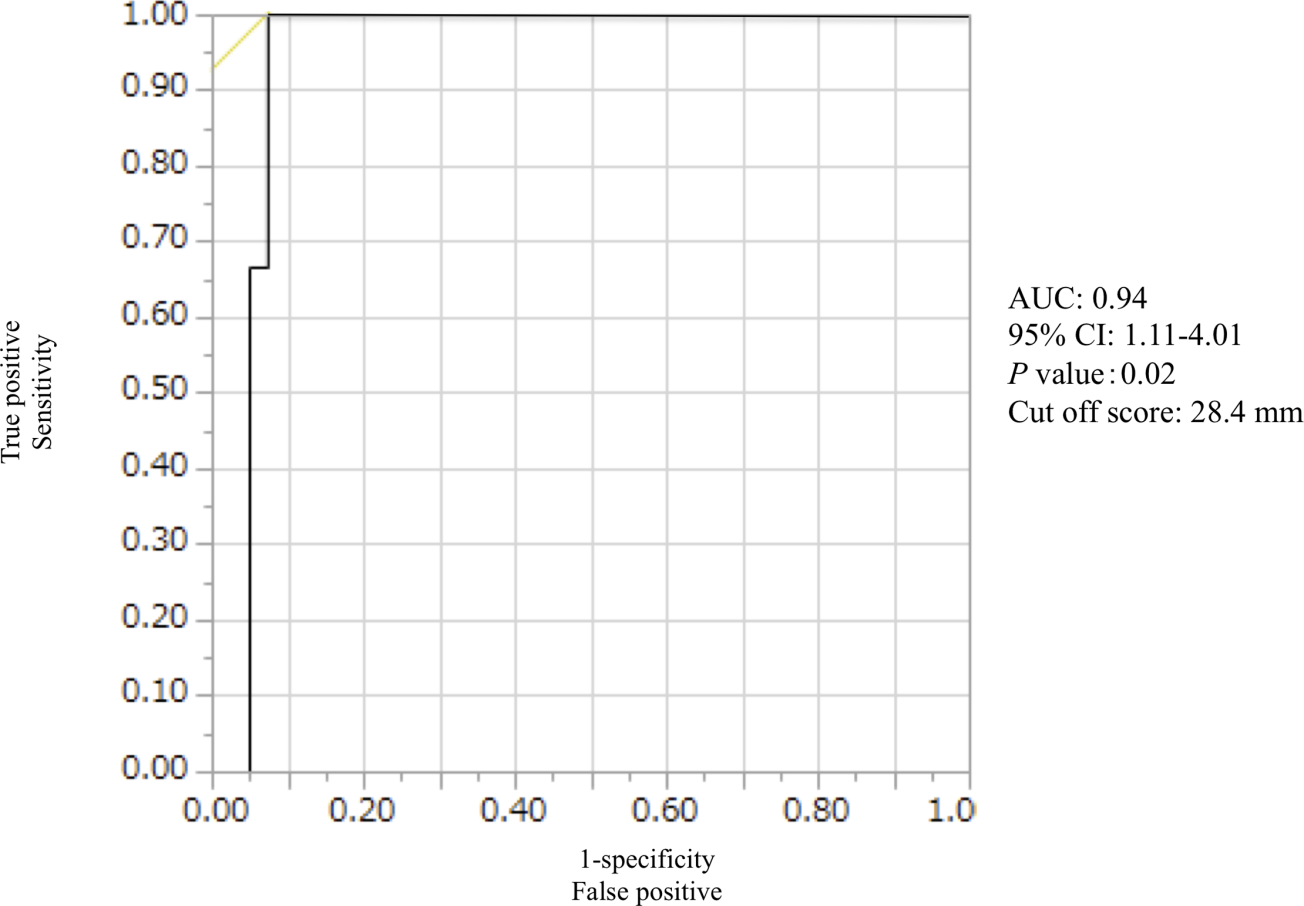
Receiver operating characteristic curve (ROC), with cut-off score for axial length to predict surgical failure in subjects who underwent internal limiting membrane peeling. The area under the ROC curve was 0.94 and the cut-off score for axial length was 28.4 mm.

Fig 3 shows the ROC curve for the ability of axial length to predict surgical failure in group 2. The AUC was 0.94 and the cut-off score for axial length was 28.4 mm.

Axial length was not correlated with postoperative VA in either group (group 1: *P* = 0.57 and group 2: *P* = 0.36; S1 Fig).

## Discussion

We set out to evaluate the anatomical closure rate and visual outcome in patients undergoing MIVS for MH with either ILM flap inversion or conventional ILM peeling. Though preoperative characteristics were similar in both groups, the MH closure rate was significantly higher after ILM flap inversion than after ILM peeling. A multiple regression analysis revealed that MH diameter was an independent factor predicting 6-month postoperative VA in both groups. Furthermore, logistic regression analysis revealed that long axial length was an independent factor indicating an unclosed MH after ILM peeling.

The ILM flap inversion technique was first introduced to treat large MHs, and modifications were then developed to allow vitreous surgeons to more easily achieve a similar effect.[14–18] Currently, various techniques are used to cover the MH or pack the ILM into the MH. The healing process in the MH after ILM flap inversion remains unclear, but postoperative OCT findings suggest that the process may be characterized by the early appearance of a hyper-reflective material and/or ILM bridge in the MH, which functions as a scaffold for tissue proliferation. Our experience confirms that this material gradually diminishes and finally becomes insignificant, while the foveal surface becomes smooth and the MH closes completely. These changes may be caused by the inverted ILM flap tissue inducing glial cell proliferation, which then fills the MH and enhances closure.[8] However, though almost all MHs can be closed with this novel technique, the fovea loses its original stratified structure in many cases, and the outer layers, i.e., the IS/OS and ELM, remain relatively homogenous. In the current study, we found that these outer layers gradually returned, but not in all cases, and that there were no significant differences in structural changes over time after ILM flap inversion or conventional ILM peeling. This finding may explain the limitations in visual recovery that we also observed after ILM flap inversion.

Historically, MH diameter is closely associated with a successful anatomical outcome and postoperative VA.[5–7] Though the impact of ILM flap inversion for MH on postoperative VA requires further study, our findings add weight to previous reports suggesting that postoperative VA significantly improves after ILM flap inversion.[8, 10, 17, 19] Nevertheless, even though ILM peeling unfortunately resulted in three unsuccessful outcomes in the current study, there was no significant difference in 6 month-postoperative VA between the groups, despite previous reports on the advantages of ILM flap inversion.[8] Furthermore, multiple regression analysis showed that MH diameter was an independent factor predicting 6-month postoperative VA in both groups, suggesting that although ILM flap inversion can close even large MHs, it does not lead to better visual outcomes than ILM peeling. This may be because the improvements in foveal structure after ILM flap inversion do not include the restoration of the normal layered structure of the retina.

The most important finding in the current study was that axial length and MH diameter were both independent factors predicting 6-month postoperative BCVA after ILM peeling in a multiple regression analysis. Furthermore, a logistic regression analysis showed that axial length (OR = 2.12) was the only independent factor predicting surgical failure after ILM peeling, and that this predictive ability was very high (AUC: 0.94), with a cut off score of 28.4 mm. This can be interpreted as meaning that the OR for MH surgical failure approximately doubles for each 1 millimeter increase in axial length. On the other hand, MH diameter had no ability to predict surgical failure. These results resemble those of previous reports that found that ILM flap inversion contributed to a high MH closure rate in patients with high myopia and no retinal detachment.[10, 11, 20] A long axial length can contribute to macular retinoschisis, which can develop into MH and finally result in MH retinal detachment. Thus, we consider that the absolute value of axial length, as opposed to measurements of refractive error, should be a key criterion in choosing ILM flap inversion rather than the conventional technique. Previously, reports by vitreous surgeons have described different criteria for axial length, ranging from more than 26.5 mm[9, 11] to 30.0 mm,[20]. Thus, there has not yet been a definitive report on the ideal axial length cut-off to indicate ILM flap inversion. We are the first to report a clear cut-off value of 28.4 mm, but the axial lengths of the three eyes in our study with unsuccessful outcomes after ILM peeling were 29.43 mm, 29.41 mm, and 28.39 mm. MH diameter in the first two cases was 424 μm and 872 μm, respectively, while in the final case it was 366 μm. Thus, based on our current results, we recommend the use of the ILM flap inversion technique to treat MH in eyes with an axial length ≥ 28.0 mm, regardless of MH diameter, in order to ensure postoperative MH closure.

The ILM flap inversion technique presents technical difficulties depending on the surgeon’s skill. In particular, it is important to prevent the ILM flap from detaching from the macula. Our experience suggests that the ILM flap should be inverted during the last stages of surgery, just before fluid-air exchange, and that fluid-air exchange should be performed as gently as possible, with low fluid aspiration and air infusion pressure. Nevertheless, we believe that our results, which were based on the work of an experienced vitreous surgeon who avoided the use of any adjuvant dyes, and our data, which was derived from a multivariate analysis, may form the basis for new criteria for selecting patients best suited to undergo ILM flap inversion to treat MH without retinal detachment.

The limitations of this study included a relatively short follow-up time of 6 months, a relatively small study population of 90, the fact that all surgeries were performed by the same surgeon, and the omission of postoperative findings for subjective retinal sensitivity, such as automated standard perimetry, or objective retinal function, such as focal electroretinography. Moreover, the rate of long-standing MH was higher in group 1 than in group 2. An additional potential limitation was the very high standard deviation for MH duration in both groups, but this was due in both cases to single outlier patients with extremely long MH duration (121 months for a subject in group 1 and 243 months for a subject in group 2). The multiple statistical tests used in this study may also have caused a multiplicity issue, and in the future, we hope to perform a follow-up prospective study with a longitudinal mixed-effects model with main effects and group-by-factor interaction, as well as a larger, multi-center study population. Such a follow-up study would confirm our conclusion on indicating the ILM flap inversion technique based on axial length. Until then, we cannot conclude that there is a large difference in the outcome of the two techniques. Finally, ILM removal is a procedure that removes a normal part of the retina. The long-term functional outcomes of this technique remain unclear, although there are reports that microperimetry and other functions may be affected.[21, 22]

In conclusion, this study showed that ILM flap inversion resulted in a significantly higher surgical success rate for MH than ILM peeling. A multiple regression analysis revealed that MH diameter was an independent factor predicting 6-month postoperative VA, regardless of the surgical procedure chosen, and a logistic regression analysis revealed that axial length was the only independent factor indicating surgical failure after ILM peeling. Thus, we recommend MIVS with ILM flap inversion for MH, particularly in patients with high myopia and an axial length ≥ 28.0 mm.

## Supporting information

S1 Fig Relatinship between axial length and 6M-postoperative VA. Axial length was not correlated with postoperative VA in either group (group 1: R = 0.13, P = 0.57 and group 2: R = 0.17, P = 0.36).
(TIFF)
