# Supplementary figures and images for: Surgical results of internal limiting membrane flap inversion and internal limiting membrane peeling for macular hole
Source: PLoS One. 2018 Sep 13;13(9):e0203789. doi: 10.1371/journal.pone.0203789 (PMC6136763; doi:10.1371/journal.pone.0203789)

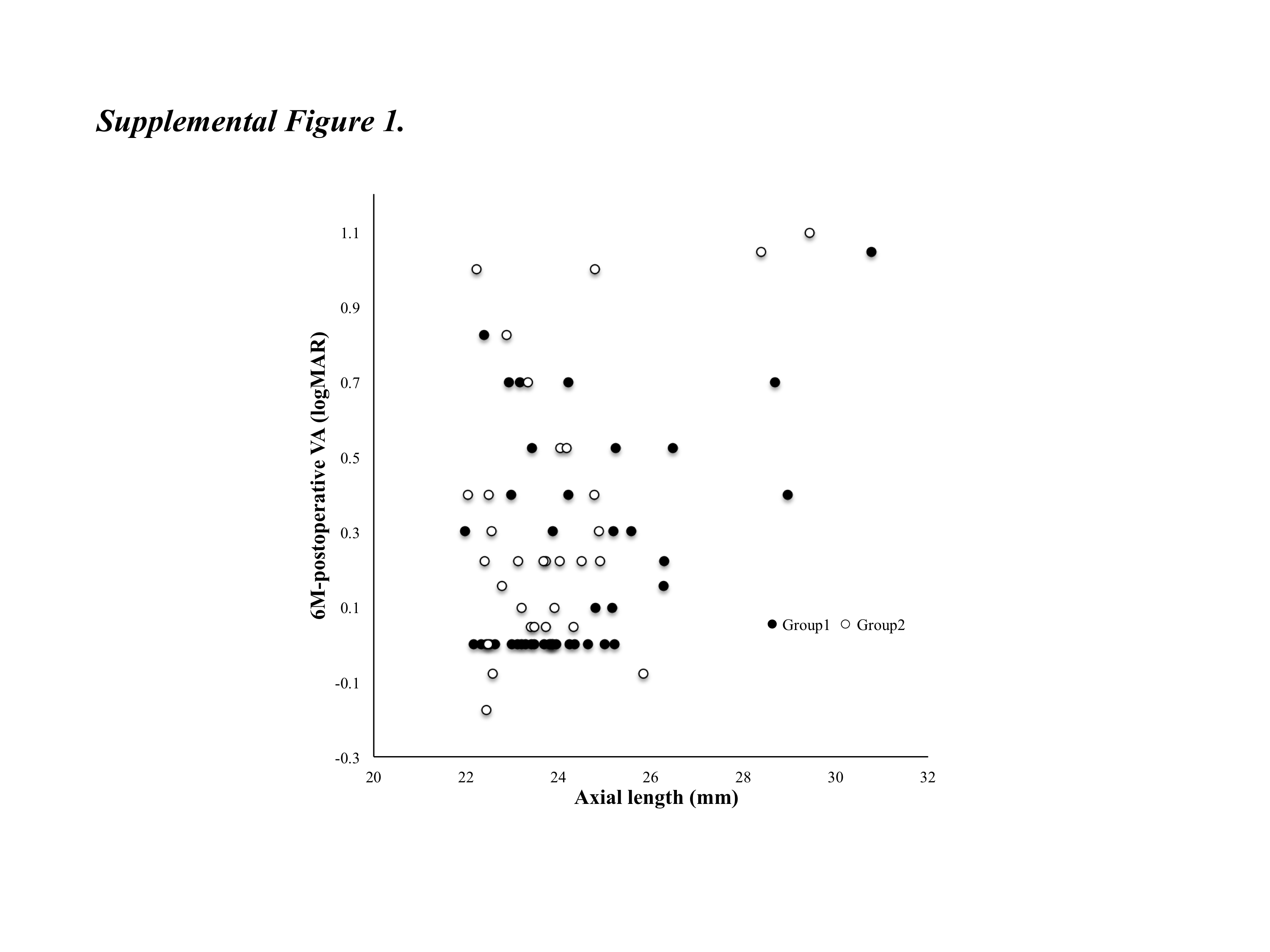

Supplement: S1 Fig — Axial length was not correlated with postoperative VA in either group (group 1: R = 0.13, P = 0.57 and group 2: R = 0.17, P = 0.36). (TIFF) [file pone.0203789.s001.tiff]
